# The complete chloroplast genome sequence of an conifer plant *Torreya grandis* (Pinales, Taxaceae)

**DOI:** 10.1080/23802359.2018.1522975

**Published:** 2018-10-08

**Authors:** Zhen-Peng Miu, Jin-Ming Zhang, Jian-Hui Li, Xin Hong, Tao Pan

**Affiliations:** a Anhui University, Hefei, China;; b Anhui Normal University, Wuhu, China;; c Quzhou Academy of Agricultural Sciences, Quzhou, China;; d Guangxi Key Laboratory of Plant Conservation and Restoration Ecology in Karst Terrain, Guangxi Institute of Botany, Guangxi Zhuang Autonomous Region and Chinese Academy of Sciences, Guilin, China

**Keywords:** Torreya grandis, Chloroplast genome, Taxaceae, phylogeny

## Abstract

Chinese Nutmeg Tree (*Torreya grandis*) is a species of conifer in Taxaceae, which has a wide range in eastern and south-eastern China. *Torreya grandis*, endemic to eastern and south-eastern China, is a large tree, which own important economic value. Here, we report the complete chloroplast (cp) genome sequence and the cp genomic features of *T. grandis*. The genome was 136,949 bp long with 117 genes comprising of 82 protein-coding genes, 31 tRNA genes, and 4 rRNA genes. Phylogenetic analysis suggested that *Torreya* species formed a monophyletic clade within the Taxaceae family, and *T. jackii* is at the base of the tree and *T. grandis* and *T. fargesii* are sister groups of the inner clade.

Chinese Nutmeg Tree (*Torreya grandis*) is a species of conifer in Taxaceae, which has a wide range in eastern and south-eastern China (http://www.efloras.org/). Its natural habitats are often along streams in mountains and open valleys between 200 m and 1400 m a.s.l. *Torreya grandis* is a large tree, different from *T. jackii*, which can attain height of 25 m, and possibly as high as 39 m. The seeds of *T. jackii* own high economic value due to its edible, medicinal value and produce oil. Due to the exploitation for its high economic value (timber and seeds) and general forest clearance for agricultural expansion, the population had decreased sharply in the past.

In this study, we sequenced the complete chloroplast (cp) genome and reported the cp genomic features of *T. grandis*. The sample of *T. grandis*, stored in the Herbarium of Anhui University, was collected from the Hangzhou Botanical Park in Zhejiang Province (30°15′N, 120°06′E). The sequence of *T. grandis* cp genome has been deposited in public databases (Genbank accession number: KY369757). Total genomic DNA was extracted from the fresh mature leaves of *T. grandis*, and sequenced on an Illumina Hiseq 2500 platform (San Diego, CA, U.S.A.). Genome sequences were screened out and assembled with Velvet v1.2.07 (Zerbino and Birney 2008). The total length of tung tree cp genome was determined to be 136,949 bp with the circular structure similar to other Taxaceae cp genomes. The genome is structured with 117 unique genes including 82 distinct protein-coding genes, 4 distinct rRNA genes, and 31 distinct tRNA genes. Among the protein-coding genes, 32 were distributed on the H-strand and 50 on the L-strand. In addition, there are 5 protein-coding genes duplicated. The genes coding for proteins, rRNA, and tRNA are 63792, 4515, and 2447 bp, representing 46.58, 3.30, and 1.79% of the cp genome, respectively. The base composition of cp genomes was A (32.50%), G (17.81%), C (17.60%), and T (32.04%), so the percentage of A and T (64.54%) was higher than that of G and C (35.41%). To confirm the phylogenetic position of *T. grandis* (Kang and Tang 1995; Li et al. 2001), a molecular phylogenetic tree was constructed with MEGA 6.0 (Tamura et al. 2013) based on the maximum likelihood method using a dataset of the complete genome sequences of 14 individuals of 11 species from Coniferopsida. The six species in Taxaceae were clustered together. *Torreya jackii* is at the base of the tree and *T. grandis* and *T. fargesii* are sister groups of the inner clade (Figure 1).

**Figure 1.**
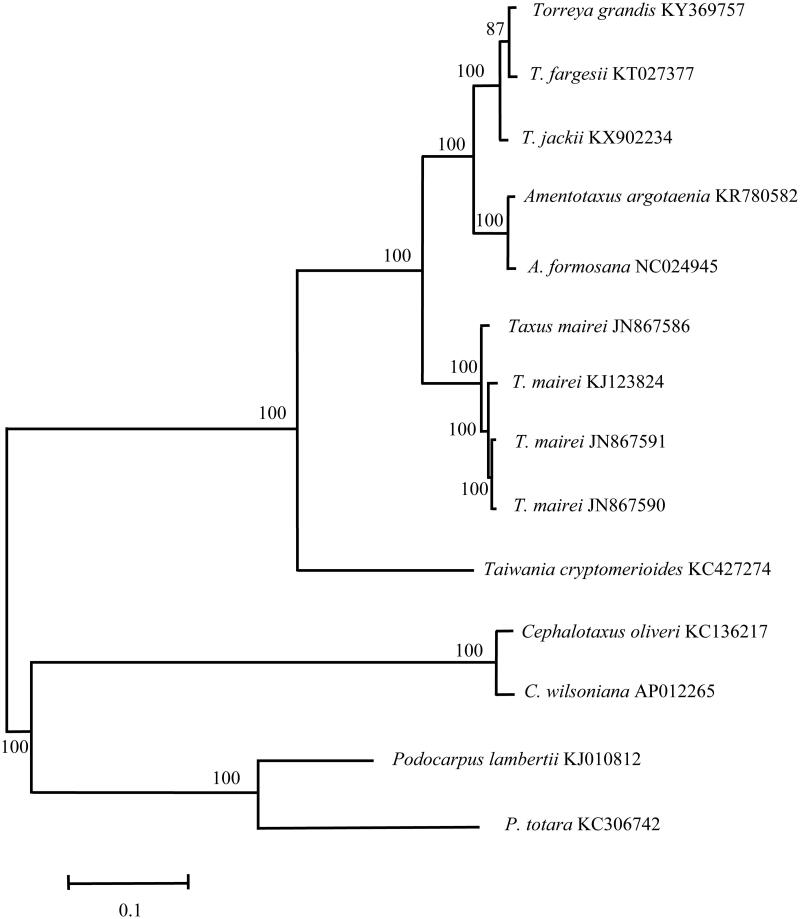
Phylogenetic position of *Torreya grandis* inferred by maximum likelihood (ML) of complete cp genome. The bootstrap values were based on 1000 replicates, and are shown next to the nodes.

In recent decades, several cp genomes of Taxaceae species have been reported, including *Amentotaxus formosana* (Hsu et al. 2014), *A. argotaenia* (Li et al. 2015), *T. jackii* (Li et al. 2017), *T. fargesii* (Tao et al. 2015), and *Taxus mairei* (Zhang et al. 2014). This newly reported chloroplast genome provides a good foundation for the identification and genotyping of Taxaceae species.
